# Supplementary material for: Cattle Cleanliness from the View of Swedish Farmers and Official Animal Welfare Inspectors
Source: Animals (Basel). 2021 Mar 27;11(4):945. doi: 10.3390/ani11040945 (PMC8066830; doi:10.3390/ani11040945)
Supplement: Supplementary file 1 [file animals-11-00945-s001.pdf]

Survey: Clean and dirty cattle (Farmers and animal welfare inspectors)

A translated short version, from Swedish.

1) The first part consisted of question concerning the farm, the animals and the checkpoint "clean enough animals". This part was mainly answered by the animal welfare inspectors.

- County board.
- Number of animals at the control point.
- Main production focus: *meat* or *milk* could be ticked.
- Type of control: *routine, suspected mistreatment, follow-up, notification from abattoir.*
- Number of animals not clean enough.
- Is the checkpoint (cattle are clean enough) considered to be *yes* or *no*.
- If *yes*, and there are animals not clean enough, what is the reason behind reporting *yes*? *Few animals not clean enough, minor non-compliance, temporary non-compliance, preventive measure directly taken, preventive measure already planned, other.*

2) The second part consisted of questions concerning reasons of why there are dirty animals, if any. This part was answered by the animal welfare inspectors in dialogue with the farmer. Several reasons of the ones below, could be stated.

- Housing conditions and management: *too long stall platforms, no stall dividers, improper stall design, too few cubicles/lying stalls, improper grouping of animals, technical failure, new animals unfamiliar with the system, crowding in pen or on deep bedded lying area, insufficient cleaning of lying areas or walking alleys, lack of bedding material, no routine for grooming dirty animals, no or insufficient coat trimming routines.*

3) The third part consisted of questions to all farmers, both farms with dirty animals and farms with clean animals. The question to farmers with dirty animals was: "W*hat would you need to do to keep your animals clean?*" and the question to farmers with clean animals was: "*How do you succeed in keeping your animals clean?*" Several reasons of the ones below, could be stated.

- *Shorter/short queues for slaughter, more/enough working hours, better/good management routines, better/good facilities, better/good economy, better/good access to bedding material, more/no lack of staff, better/good physical health, better/good mental health, other.*
